# Estrogen Abolishes Protective Effect of Erythropoietin against Cisplatin-Induced Nephrotoxicity in Ovariectomized Rats

**DOI:** 10.5402/2012/890310

**Published:** 2012-11-06

**Authors:** Zahra Pezeshki, Mehdi Nematbakhsh, Safoora Mazaheri, Fatemeh Eshraghi-Jazi, Ardeshir Talebi, Hamid Nasri, Tahereh Safari, Azam Mansouri, Farzaneh Ashrafi

**Affiliations:** ^1^Water and Electrolytes Research Center, Isfahan University of Medical Sciences, Isfahan 81745, Iran; ^2^Department of Physiology, Isfahan University of Medical Sciences, Isfahan 81745, Iran; ^3^Kidney Diseases Research Center, Isfahan University of Medical Sciences, Isfahan 81745, Iran; ^4^Department of Clinical Pathology, Isfahan University of Medical Sciences, Isfahan 81745, Iran; ^5^Department of Internal Medicine, Isfahan University of Medical Sciences, Isfahan 81745, Iran

## Abstract

*Introduction*. Nephrotoxicity is one the side effect of cisplatin therapy and erythropoietin has been candidate as a nephroprotectant agent. However, its nephroprotective effect when it is accompained with estrogen has not been studied in female. *Methods*. 27 ovariectomized female Wistar rats divided into five groups. Groups 1 & 2 received estradiol valerate (0.5 mg/kg/week) for four weeks, and single dose of cisplatin (7 mg/kg, ip) was administrated at the end of week 3. Then the group 1 was treated with erythropoietin (100 U/kg/day), and the group 2 received vehicle during week 4. Groups 3 and 4 were treated similar to group 1 and 2, except for placebo instead estradiol valerate. Group5 (negative control) received placebo during the study. Animals were killed at the end of week 4. *Results*. In non-erythropoietin treated rats, cisplatin significantly increased the serum levels of blood urea nitrogen and creatinine (*P* < 0.05). However, these biomarkers significantly decreased by erythropoietin (*P* < 0.05). The weight loss, kidney weight, and kidney tissue damage score in rats treated with cisplatin but without estradiol were significantly less than the values in similar group when estradiol was present (*P* < 0.05). *Conclusion*. It seems that erythropoietin could protect the kidney against cisplatin-induced nephrotoxicity. This protective effect was not observed when estrogen was present.

## 1. Introduction

Cisplatin (*cis*-diamminedichloroplatinum II, CP) as a potent antitumor drug is commonly used for a wide variety of tumors, including head and neck, lung, testis, ovary, and breast tumors [1]. However, it has many side effects like ototoxicity, gastrotoxicity, myelosuppression, and allergic reactions. The main dose-limiting side effect of CP is nephrotoxicity [2].

CP injection leads to accumulation of platinum within kidney tissue and influences renal tubular function [3]. The renal dysfunction following exposure to CP is involved in tubular epithelial cell toxicity, apoptosis, vasoconstriction in the renal microvasculature, proinflammatory effects, and activation of mitogen-activated protein kinases [4, 5]. These events lead to wasting of sodium, potassium, magnesium, elevation in serum levels of creatinine (Cr) and blood urea nitrogen (BUN), reduction in serum albumin, and decrease in the glomerular filtration rate [2, 3, 5]. Many agents such as vitamins C and E, losartan, and l-arginine have been proposed to protect the kidney against nephrotoxicity of platinum drugs [6–8]. EPO is one of these agents used for treatment of anemia and acute renal failure induced by CP [9, 10]. EPO has antiapoptotic, antioxidant, and anti-inflammatory effects [10], and it has been used as a nephroprotective against various kidney injuries such as kidney damage induced by ischemia-reperfusion [11, 12] CP-induced nephrotoxicity [9, 13–16], and gentamycin-induced kidney toxicity [17].

EPO is a glycoprotein hormone, primarily produced by renal cortical and outer medullary fibroblasts in response to hypoxia [18]. EPO receptors (EPOR) have been identified in a large range of cell types, including proximal tubular epithelial cells, mesangial cells, renal cell carcinomas, prostatic cells, breast cancer cells, chorioallantoic membrane, uterine adenocarcinomas, and ovarian carcinomas [18]. EPOR activation leads to activation of some signaling pathways that enhance cell proliferation and mediate renoprotection [9, 11]. As mentioned before, EPO improves CP-induced acute renal failure and leads to recovery after tubular damage [9]. Experimental evidence suggests that estradiol inhibits production of EPO in female rats, when rats have exposed to various intensities of hypoxia, which is confirmed by production lower amounts of EPO in normal females than normal males. So, ovariectomized rats show a response equal to males [19], and estradiol decreases EPO gene expression during hypoxia [20]. Accordingly, the protective roles of EPO against oxidative stress may change when estradiol is accompanied by EPO. Therefore, this study was designed to find the protective role of EPO in a CP-induced nephrotoxicity when it is accompanied by estrogen.

## 2. Materials and Methods

### 2.1. Animals

The investigation was performed on 27 adult female Wistar rats (152.02 ± 2.847 g) (Animal Centre, Isfahan University of Medical Sciences, Isfahan, Iran). The rats were housed at a temperature of 23–25°C. They had free access to water and rat chow, and they were acclimatized to this diet for at least one week prior to the experiment. The experimental procedures were in advance approved by the Isfahan University Medical Sciences Ethics Committee.

### 2.2. Experimental Protocol

The animals were anesthetized by ketamine (75 mg/kg, i.p.). An incision was made in the abdominal middle line to expose and remove the ovaries from retroperitoneal space. The skin was closed with sutures. One week after the operation and recovery, the rats were allowed to acclimatize to the same diet at least for one week. Then, they were randomly divided into five experimental groups. Group 1 received estradiol valerate (500 *μ*g/kg/week) in sesame oil intramuscularly for four weeks. At the end of week 3, group 1 (*n* = 6) received single dose of CP (7 mg/kg) and then treated with EPO (100 IU/kg, i.p.) every day during week 4.

Group 2 (*n* = 5) received the same regimen as group 1, except for vehicle instead of EPO. This group was considered as the positive control for group 1. Group 3 was treated similar to group 1, except for sesame oil alone instead of estradiol valerate in sesame oil for four weeks. Then, they received single dose of CP and treated with EPO every day during week 4. Group 4 (*n* = 5) was treated with the same regimen as group 3 except for vehicle instead of EPO. This group was considered as the positive control for group 3. The negative control group (group 5) received vehicle alone during the study. In summary, the animals in group 1 were ovariectomized and were treated by estradiol valerate in sesame oil, CP, and EPO (named OVE + CP + EPO); group 2 received vehicle instead of EPO (named OVE + CP). The animals in group 3 were ovariectomized and received sesame oil, CP, and EPO (named OV + CP + EPO), while animals in group 4 were treated by vehicle instead of EPO (named OV + CP). Group 5 received only saline during the study (named OV). 

All the animals sacrificed seven days after injection of CP and taking blood samples. CP (*cis*-Diammineplatinum (II) dichloride, code P4394), EPO, and estradiol valerate were purchased from Sigma (St. Louis, MO, USA), Janssen-Cilag (Czech Republic), and Aburaihan (Tehran, Iran), respectively. The serum was collected from each blood sample and stored at −20°C until measurement. The animals' body weight was recorded daily. At the end of the experiment, the kidney and uterus were removed and immediately weighted.

### 2.3. Measurements

The levels of serum Cr and BUN were determined using quantitative diagnostic kits (Pars Azmoon, Iran). The serum level of nitrite (stable NO metabolite) was measured using a colorimetric assay kit (Promega Corporation, USA) that involves the Griess reaction. The serum level of malondialdehyde (MDA) was determined by thiobarbituric acid (TBA) 0.67% and trichloroacetic acid (TCA) 10%.

### 2.4. Histopathological Procedures

The removed kidneys were fixed in 10% neutral formalin solution and were embedded in paraffin for histopathological staining. Hematoxylin and eosin stain was applied to examine the tubular atrophy, cast, debris, and necrotic materials in the tubular lumen. Lymphocytes in interstitial tissue were considered as tubular damage. Tubular lesions were scored from 1 to 4 based on the damage intensity, where score zero was assigned to normal tubules without damage. 

### 2.5. Statistical Analysis

Data are expressed as mean ± SEM. One way ANOVA was applied to compare the weight loss, kidney weight, uterus weight, and serum levels of BUN, Cr, MDA, and nitrite between the groups. The pathological damage score of the groups was compared by the Mann-Whitney and Kruskal-Wallis tests. To determine the correlation between kidney weight and pathological damage score, the nonparametric Spearman correlation test was used. *P* values <0.05 were considered statistically significant.

## 3. Results 

### 3.1. BUN, Cr, and Nitrite Serum Levels and Uterus Weight

**Figure 1:**
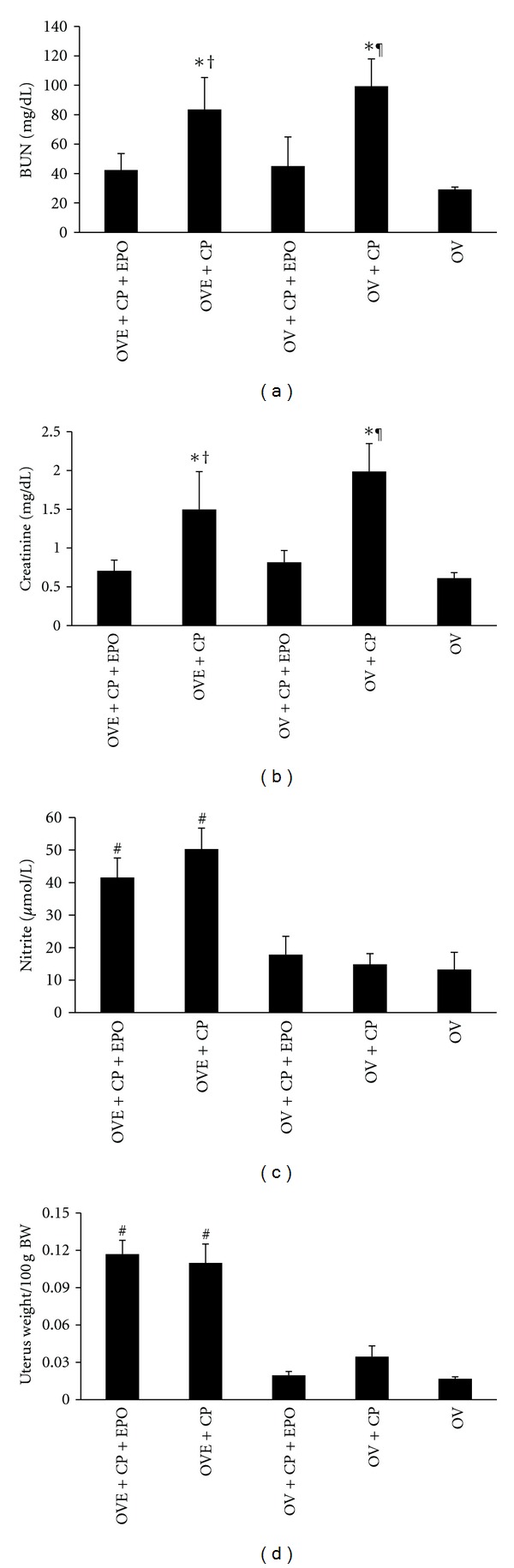
Serum levels of BUN, Cr, nitrite, and uterus weight in five experimental groups. The signs stand for significant difference (∗) from OV group; (†) from OVE + CP + EPO group; (¶) from OV + CP + EPO group; or (#) from OV + CP + EPO, OV + CP, and OV groups (*P* < 0.05).

CP-induced nephrotoxicity was approved by the increase of BUN and Cr serum levels in positive control groups (groups 2 and 4) when compared with the negative control group (group 5) (*P* < 0.05). Such findings were not obtained in EPO-treated groups (groups 1 and 3). In other words, EPO reduced the serum levels of BUN and Cr in ovariectomized animals treated with CP either estradiol was present or not (Figure 1). As expected, the serum level of nitrite significantly increased in estrogen-treated groups and was statistically different when compared with nonestradiol-treated group (*P* < 0.05) (Figure 1). At the end of the experiment, the uterus weight in estradiol-treated groups (groups 1 and 2) was significantly greater than others groups so, administration of estradiol was effective for the animals (Figure 1). 

### 3.2. Serum Level of MDA and Body Weight Changes, KW, and KTDS

**Figure 2:**
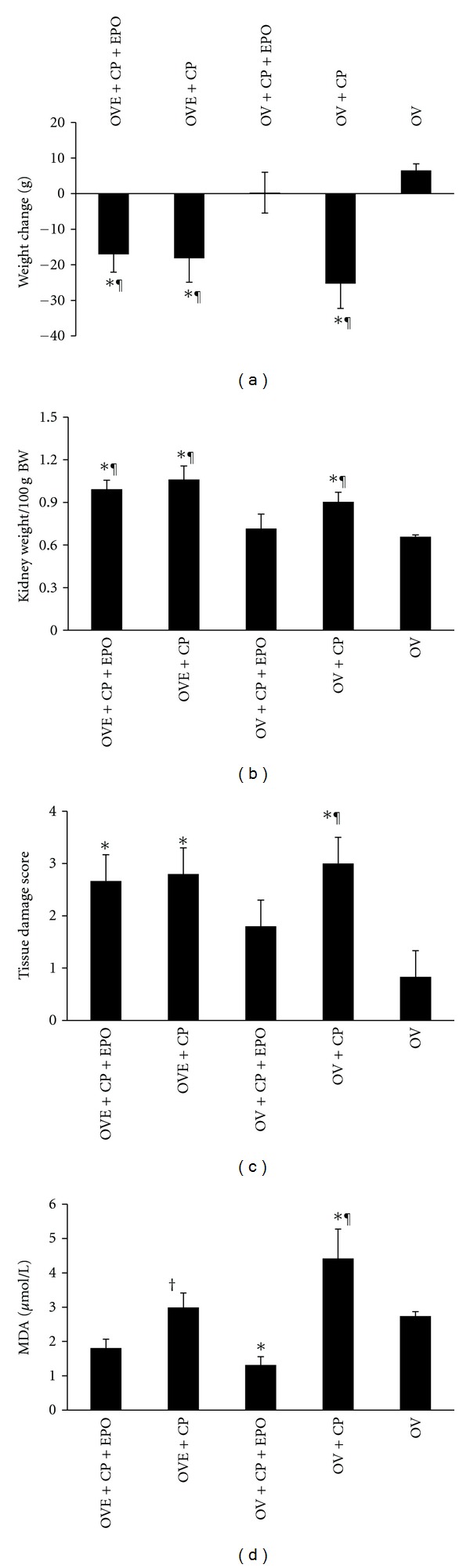
Serum level of MDA, body weight change, KW, and KTDS in five experimental groups. The signs stand for significant difference (∗) from OV group; (†) from OVE + CP + EPO group; (¶) from OV + CP + EPO group; or (#) from OV + CP + EPO, OV + CP, and OV groups (*P* < 0.05).

**Figure 3:**
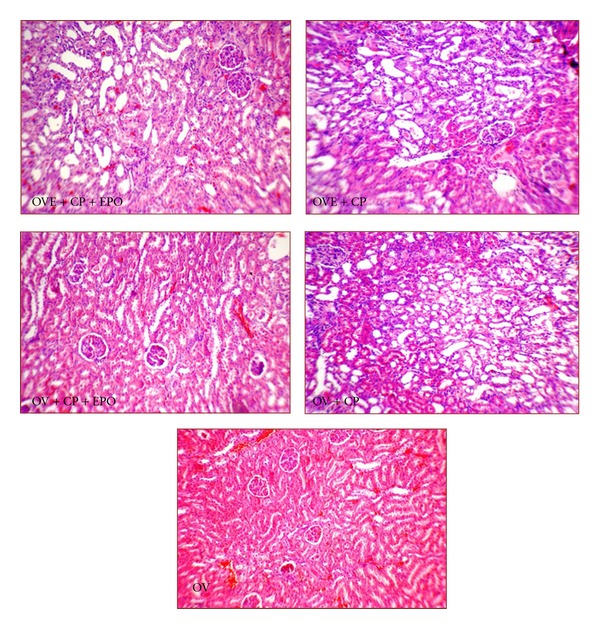
Kidney tissue images (magnification ×100). More tissue damages were observed in OVE + CP + EPO, OVE + CP and OV + CP groups.

Administration of CP increased the serum level of MDA in groups 2 and 4. However, only in group 4, the serum level of MDA was significantly different from the negative control group (*P* < 0.05). The rats treated with EPO had a lower serum level of MDA compared with the positive control groups (*P* < 0.05). Weight loss during the last week of the experiment was compared between CP injection day as the first day and seven day after CP injection as the last day of the week. The data indicates a significant weight loss in CP-treated groups 1 (OVE + CP + EPO), 2 (OVE + CP), and 4 (OV + CP) when compared with the negative control group (*P* < 0.05). This is while such weight loss was not observed in group 3. The weight change in group 3 in comparison with the negative control group was not significant, while group 3 was significantly different from other groups in this respect (*P* < 0.05) (Figure 2). The KTDS and KW of groups 1 (OVE + CP + EPO), 2 (OVE + CP) and 4 (OV + CP) increased significantly when compared with the negative control group (*P* < 0.05). However, no significant differences in mentioned parameters were detected between group 3 (OV + CP + EPO) and the negative control group (Figure 2). This indicates that EPO could protect the kidney from toxicity induced by CP, but this protection was abolished when EPO was accompanied by estradiol. The KTDS and KW in group 3 were statistically less than the corresponding positive control group. A significant correlation was detected between KW and KTDS (*r* ^2^ = 0.6304, *P* < 0.01). Samples of kidney tissue images from each group are demonstrated in Figure 3.

## 4. Discussion

The main objective of this study was to determine the protective role of EPO against CP-induced nephrotoxicity at the presence of estradiol on ovariectomized rat model. Our findings indicate that EPO reduces the serum levels of BUN, Cr, and MDA that were increased by CP. However, when estradiol was not present, higher KTDS, KW, and weight loss, that were induced by administration of CP, were reduced significantly by EPO, when compared to the positive control group. We also found a statistically significant correlation between KTDS and KW. This finding is in agreement with results of other studies [7, 21]. 

EPO may protect the kidney from acute renal damage, as its receptors are expressed in the kidney [22]. It was reported that EPO ameliorates CP-induced nephrotoxicity [9, 10, 15, 23–26], while female sex hormone, estrogen, inhibits EPO production in female rats [19] and decreases EPO gene expression during hypoxia [20]. Some evidence also has reported the sex difference response to CP-induced nephrotoxicity and renal function [27, 28]. 

It seems that although estrogen acts as a cardiovascular protectant in women before menopause, its protective role in CP-induced nephrotoxicity is failed. Estrogen enhances oxidative stress in the kidney [29] and promotes kidney toxicity in the tubules [30, 31]. Estrogen also enhances the serum level of NO as seen in our study [32, 33], and on the other hand, NO is involved in CP-induced nephrotoxicity [34, 35]. Therefore, enhancement of serum level of both estradiol and NO potentially may promote the intensity of nephrotoxicity.

In addition, EPO is affected by estrogen, too. Estrogen decreases hypoxic induction of plasma EPO, and renal EPO gene expression is mediated by increasing NO production [20, 36], and NO can reduce EPO gene expression in kidneys [37]. EPO, as an antioxidant and antiapoptotic agent, has a protective effect against CP-induced nephrotoxicity [14, 23]. Recombinant human EPO reduces the serum levels of MDA and glutathione, induced by CP treatment [38]. EPO also restores the serum levels of Cr and BUN increased by CP [14]. It also reduces the cell apoptosis by upregulation of antiapoptotic protein expression [23]. Therefore, it seems that the protective role of EPO in CP-induced nephrotoxicity is not related to its hematopoietic effect [39].

## 5. Conclusion 

We could hypothesize that the protective role of EPO may not only be attenuated by estrogen due to the effect of sex hormone on EPO [40, 41], but also estrogen itself promotes the toxicity intensity via NO production [32–35] or other pathways. This study for the first time suggests a need to pay special attention to CP therapy in women who are under estrogen replacement therapy. Of course more studies are needed to define the exact mechanism.
